# Applications of ^19^F-NMR in Fragment-Based Drug Discovery

**DOI:** 10.3390/molecules21070860

**Published:** 2016-07-16

**Authors:** Raymond S. Norton, Eleanor W. W. Leung, Indu R. Chandrashekaran, Christopher A. MacRaild

**Affiliations:** Medicinal Chemistry, Monash Institute of Pharmaceutical Sciences, Monash University, Parkville 3052, Australia; eleanor.leung@monash.edu (E.W.W.L.); indu.chandrashekaran@monash.edu (I.R.C.); chris.macraild@monash.edu (C.A.M.)

**Keywords:** fragment-based drug design, ^19^F-NMR, labelling, chemical shift, linewidth, ligand, protein, peptide

## Abstract

^19^F-NMR has proved to be a valuable tool in fragment-based drug discovery. Its applications include screening libraries of fluorinated fragments, assessing competition among elaborated fragments and identifying the binding poses of promising hits. By observing fluorine in both the ligand and the target protein, useful information can be obtained on not only the binding pose but also the dynamics of ligand-protein interactions. These applications of ^19^F-NMR will be illustrated in this review with studies from our fragment-based drug discovery campaigns against protein targets in parasitic and infectious diseases.

## 1. Introduction

With a decline in the number of new FDA-approved drugs reaching the clinic each year [1], in particular in the area of new antibiotics [2], new approaches to the development of therapeutic leads are needed. Fragment-based drug discovery (FBDD) offers one particularly promising way forward. In this article we outline some of the steps in a typical FBDD campaign, then focus on the different ways in which ^19^F-NMR can be applied in this field.

The initial phase of FBDD employs one or more sensitive biophysical techniques to detect the binding of small molecular entities (known as fragments) to a target protein. Indeed, the development of biophysical techniques with sufficient sensitivity and throughput to enable fragment screening has been one of the innovations that has enabled FBDD to expand.

FBDD campaigns identify small ligands (‘fragments’), which, because of their small size (typically < 300 Da), tend to bind with relatively low affinity, and then develop these into larger, higher-affinity ligands [3,4]. The major advantage of FBDD over more traditional high-throughput screening is that FBDD provides a more rapid and effective means of identifying ligands for a protein target. As there are fewer fragment-sized molecules than lead- or drug-sized molecules, FBDD samples chemical space more efficiently than traditional approaches and therefore requires fewer compounds to be tested to identify suitable hits as starting points for development. Furthermore, there is some evidence to suggest that the lead compounds that emerge from FBDD have better physicochemical properties (described by Lipinski’s ‘rule of five’ [5]) than those from traditional drug discovery approaches, and are therefore more likely to result in orally bioavailable leads [6].

A recent analysis of compounds that have emerged from FBDD programs on the Practical Fragments blog hosted by Dan Erlanson [7], lists more than 30 fragment-derived compounds in various stages of clinical evaluation, while acknowledging that this number is likely to be an underestimate. Indeed, the recent approvals of vemurafenib, a B-Raf (V600E) inhibitor developed by Plexxikon (Berkeley, CA, USA) for late-stage melanoma [8], and venetoclax (ABT-199), which inhibits the interaction of Bcl-2 with its protein partners [9,10], validate FBDD as an approach to support the development of clinically useful drugs [11]. Moreover, FBDD has a demonstrated ability to develop inhibitors of protein-protein interactions (PPIs) [12], which have traditionally been regarded as challenging drug targets by the pharmaceutical industry.

A successful FBDD program requires a biomolecular target that is relatively stable and can be produced in milligram quantities, a well-constructed fragment library [13], one or more robust biophysical screening methods [14], and access to medicinal chemistry expertise to develop promising hits. A high-resolution structure of the target, determined by either X-ray crystallography or NMR spectroscopy, is a significant advantage. Equally important is high-quality structural information on the binding pose(s) of elaborated fragments as this is required to guide medicinal chemistry optimization. While this information can be obtained using X-ray crystallography [15,16], there are numerous examples where visualization of the bound ligand in this way is not feasible because crystallization of the ligand-target complex is too slow or fails completely. In these instances, alternative methods are required, and we believe that ^19^F-NMR is a valuable approach. Some of the attributes of ^19^F that favour its application are that it is a spin ½ nucleus, it is the second most sensitive stable NMR-active nucleus, with a sensitivity of 83.4% relative to ^1^H, and it is the 100% naturally abundant isotope. This favourable intrinsic sensitivity is effectively enhanced by the absence of a natural background of fluorine in most biological samples. Moreover ^19^F resonances are highly sensitive to chemical environment, with a chemical shift range spanning several hundred ppm.

## 2. Screening Fragment Libraries

Fragment screening is typically undertaken using biophysical techniques because conventional biochemical methods are generally not sufficiently sensitive to identify the modest (typically mM) affinity of fragments for the target protein. Many such techniques are used to identify fragment hits, each with its own inherent strengths and weaknesses [14], and it is advisable to employ at least two orthogonal methods to avoid false positives. These methods include ligand-detected NMR, protein-detected NMR (e.g., two-dimensional HSQC), surface plasmon resonance (SPR), X-ray crystallography, isothermal titration calorimetry (ITC) and thermal shift assays [14].

^19^F-NMR can already contribute at this initial stage of a FBDD campaign. Dalvit and co-workers had demonstrated the value of ^19^F-NMR as a probe of ligand binding in traditional drug development campaigns [17], and in 2009 they described a fluorinated fragment library that took into account the local environment of fluorine. This library, designated LEF (Local Environment of Fluorine), combined with ^19^F-NMR ligand-based screening, proved to be an efficient and sensitive approach for hit identification and for probing the presence of ‘fluorophilic protein environments’ (i.e., potential fluorine binding sites on a protein surface) [18]. Further applications of ^19^F-NMR screening in drug development, and in particular the relationship between ^19^F chemical shifts and the nature of fluorine–protein interactions, were described subsequently [19]. The same group also described an interesting variant of these screening approaches in which the enzymatic conversion of a fluorine-containing substrate to product was monitored by ^19^F-NMR; this was exemplified with the membrane-bound enzyme fatty acid amide hydrolase, using a library of fluorinated fragments to inhibit the enzymatic conversion [20].

Jordan and co-workers at Amgen (Thousand Oaks, CA, USA) have also evaluated ^19^F-NMR-based fragment screening and shown that it is not only a rapid and sensitive method for detecting fragment hits, but can also contribute to the development of structure-activity relationships (SAR) on the hit-to-lead path and provide an efficient means of assessing target druggability [21]. They also noted that the large chemical shift dispersion and narrow linewidth (1–2 Hz in the presence of proton decoupling, although we note that proton decoupling is often not used) for ^19^F resonances of the free ligands enable the screening of a large number of compounds in a cocktail without the complication of signal overlap. ^1^H-based NMR fragment screening is typically performed in cocktails of 5–6 compounds, whereas their ^19^F screening cocktails contained 12–13 compounds, with no need for any type of specific pooling strategy, and they suggested that this number could be safely extended to ≥20 compounds per cocktail.

The utility of ^19^F-based fragment screening was demonstrated recently with the identification of a second-site fragment through ^19^F-NMR screening that binds to a specific pocket of the aspartic acid protease, β-secretase (BACE-1). The identification of this second-site fragment via an inter-ligand NOE experiment enabled a fragment-linking approach, which ultimately yielded a molecule exhibiting a >360-fold increase in potency while maintaining reasonable ligand efficiency [22].

^19^F-NMR can also be employed in fragment screening where the target protein is ^19^F labelled rather than the ligands. For example, Pomerantz and co-workers [23] undertook a small-molecule screen using protein-observed ^19^F-NMR with the transcription factor binding domain of the CREB binding protein (CBP)/p300, KIX. Screening of 508 compounds and validation by ^1^H-, ^15^N-HSQC identified a minimal pharmacophore for the MLL-KIX interaction site. In the next section we provide several examples of how ^19^F-NMR of ^19^F-labelled proteins can be used to characterize ligand binding by proteins that are the targets for therapeutic development using both fragment-based and structure-based approaches.

## 3. Characterizing Ligand Binding by ^19^F-NMR

In order to facilitate the elaboration of promising fragment hits identified by screening, it is essential to have simple and reliable assays that establish not only whether elaborated fragments bind to the target protein but also whether they do so via the desired binding site. ^19^F-NMR has been used extensively in studies of protein structure and interactions [24,25], and proves to be a valuable tool in FBDD campaigns as well as in probing the binding of other ligands relevant to such efforts.

### 3.1. Ligand Binding to the SPRY Domain-Containing SOCS Box Protein 2 (SPSB2)

**Figure 1:**
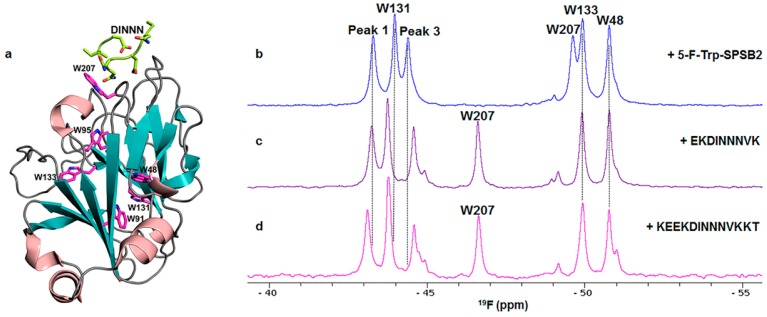
(**a**) Crystal structure of SPSB2 with bound DINNN peptide (PDB id: 3EMW). The six Trp residues of SPSB2 (W48, W91, W95, W131, W133 and W207) are shown in stick representation and coloured magenta. Four Trp resonances were successfully assigned using site-directed mutagenesis, (W48, W131, W133, and W207) but the completely buried W91 and W95 could not be assigned by this means and are labelled as peak 1 or 3. W207 is closest to DINNN; ^19^F-NMR spectra of 5-F-Trp-SPSB2 in the absence (**b**) and presence (**c**,**d**) of 9- and 13-residue peptides containing the key binding epitope from iNOS, DINNN. All ^19^F spectra were acquired at 30 °C with 100 µM 5-F-Trp-SPSB2 in 50 mM sodium phosphate, pH 7.4, 50 mM NaCl, 2 mM EDTA, 2 mM DTT, 0.02% sodium azide. The W207 resonance was significantly perturbed by DINNN-containing peptides, indicating that these peptides target the iNOS binding site [31].

The SPRY domain-containing SOCS box protein 2 (SPSB2) regulates inducible nitric oxide synthase (iNOS) in target cells such as macrophages by mediating its proteasomal degradation [26,27]. Inhibiting this interaction prolongs the intracellular lifetime of iNOS, leading in turn to enhanced killing of infectious pathogens such as bacteria and parasites. SPSB2 recognizes a linear motif (DINNN) in the disordered N-terminus of iNOS, and ligands that target the DINNN binding site on SPSB2 are potentially novel anti-infective agents [28]. We have explored ^19^F-NMR as a means of probing ligand binding to SPSB2. All six Trp residues in SPSB2 were replaced with 5-fluoro-tryptophan (5-F-Trp) by employing a Trp auxotroph strain of *Escherichia coli*. The labelled protein was correctly folded and able to bind a DINNN-containing peptide with similar affinity to that of native SPSB2. Six well-resolved 5-F-Trp resonances were observed in the ^19^F-NMR spectrum and were assigned using site-directed mutagenesis (Figure 1). The ^19^F resonance of W207 shifted downfield upon binding to DINNN-containing peptides [29]. Other resonances were perturbed to a lesser extent, although their shifts were sensitive to the composition of the peptide. Analogues of compounds identified in a fragment screen also perturbed the W207 resonance, confirming their binding to the iNOS peptide-binding site on SPSB2. ^19^F-NMR promised to be a valuable approach in developing inhibitors that bind to the DINNN binding site.

In support of this, ^19^F-NMR has been used to confirm the binding sites of cyclic peptides containing the DINNN sequence [29,30]. The ^19^F resonance of W207 shifted significantly downfield upon binding to the cyclic peptide, Ac-c[CVDINNNC]-NH_2_ [29], confirming its binding to the iNOS binding site. The extent of the chemical shift change is ~0.5 ppm less than with the 13-residue linear peptide [31], suggesting that the cyclic peptide bound slightly differently to the iNOS binding site of SPSB2. The peak of the other solvent-exposed Trp (W131) did not shift as much as in Figure 1c,d, also consistent with a slightly different binding pose for the cyclic peptide.

**Figure 2:**
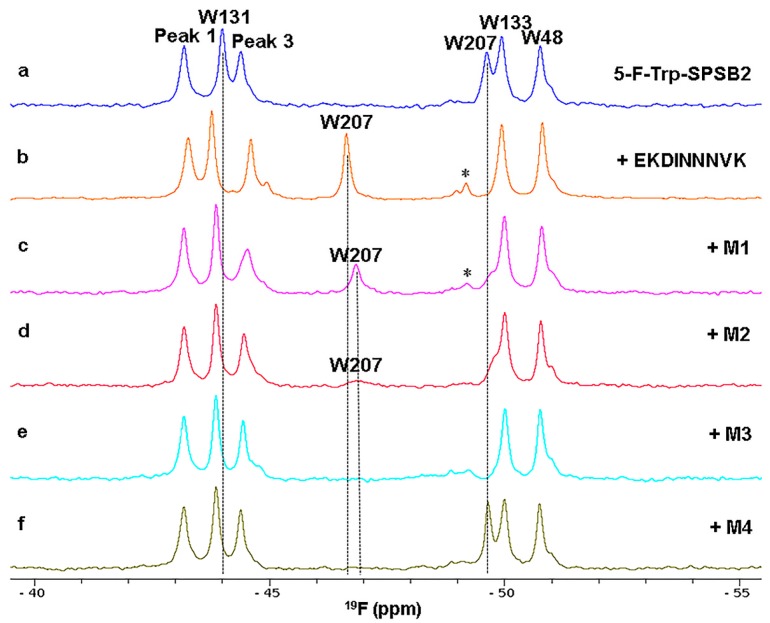
^19^F-NMR spectra of 5-F-Trp-SPSB2 in the absence and presence of peptidomimetics **M1**, **M2**, **M3** and **M4**. Chemical structures of **M1**–**M4** are shown in Figure S1. The peptide EKDINNNVK was included as a control. Each experiment contains 50 µM of 5-F-Trp-SPSB2 in the presence of either 9-residue long DINNN-containing peptide or mimetic at a SPSB2: peptide/mimetic molar ratio of 1:1.5. All six 5-F-Trp residues in SPSB2 were observed in the ^19^F-NMR spectrum. Most 5-F-Trp resonances (W48, W131, W133 and W207) were assigned except the completely buried Trp residues, W91 and W95 [31], which are labelled as peaks 1 and 3. The W207 resonance was significantly perturbed by both the peptide and the mimetics. In some instances, the presence of mimetic (e.g., **M1**–**M3**) caused the appearance of some minor peaks, which appear to be minor conformers of peak 3 and W48. The peaks marked with asterisks are from denatured protein; all six 5-F-Trp resonances converged to a single peak at −49.2 ppm when the temperature was raised to 50 °C. ^19^F-NMR spectra were recorded at 30 °C in 50 mM sodium phosphate, pH 7.4, 50 mM NaCl, 2 mM DTT, 2 mM EDTA, 0.02% sodium azide at 564 MHz on a Bruker Avance 600 spectrometer equipped with a cryoprobe tuned to ^19^F and without ^1^H decoupling.

The power of ^19^F-NMR is well illustrated by our recent studies on the development of peptide mimetics that also target the iNOS binding site on SPSB2 [32]. As shown in Figure 2, the binding of mimetics **M1** and **M2** to 5-F-Trp-SPSB2 caused similar downfield shifts of the W207 resonance to that caused by c[CVDINNNC]-NH_2_ (not shown), although the chemical shift changes were slightly larger and the peak widths were broader for **M1** and **M2** (Figure 2). Although the spectral perturbations for **M1** and **M2** were relatively similar, a sharper W207 resonance was observed with **M1** than with **M2**. In the presence of **M3**, the W207 resonance broadened beyond detection, suggesting that the complex with **M3** samples multiple conformations. In contrast, **M4** (with an expected occupancy of 99.8% based on its *K*_D_ of 442 nM) did not perturb the chemical shift of the W207 resonance, suggesting that this mimetic binds to SPSB2 in such a way that W207 is not perturbed in the same way as with the cyclic peptide and other mimetics.

To further understand the observed binding dynamics of the most potent peptidomimetic **M1**, a 1 μs molecular dynamics (MD) simulation was performed of **M1** docked with the crystal structure of SPSB2 [28]. The distance between the side chain nitrogen of W207 and one of the Asn residues of **M1** was found to fluctuate between 3.1 and 10.3 Å throughout the simulations. These observations suggest that although mimetic **M1** is bound to SPSB2, it did not lock the iNOS binding site of SPSB2 into a single bound conformation, which is consistent with the observation of a broader ^19^F resonance for W207 in the presence of **M1** compared with the cyclic peptides [29,30].

The 5 position of the Trp ring was chosen for these and other studies as 5-F-Trp resonances showed greater dispersion than 4- or 6-F-Trp (7 ppm vs. 6 ppm or 3 ppm, respectively) in studies of d-lactate dehydrogenase [25,33], and less broadening at higher magnetic fields as a consequence of chemical shift anisotropy [34]. In order to obtain high incorporation of 5-F-Trp, we have generally used a Trp auxotroph strain that grows only when Trp is present in the medium [35]. As 5-F-Trp proved to be inhibitory for growth, bacteria were first grown in LB medium until they reached mid-exponential phase, then transferred to M9 medium containing 1% Casamino acids with no Trp. After 1 h shaking, 5-F-Trp was added to the medium and, after a further 30 min, expression of the target protein was induced by addition of IPTG.

In the case of SPSB2, the use of a Trp auxotroph gave a higher yield of labelled protein than an alternative method using a non-auxotrophic BL21 (DE3) bacterial strain, with glyphosate added prior to IPTG induction in order to block the biosynthesis of aromatic amino acids. Generally, the toxicity of glyphosate affects cell growth, and thus the overall expression level, but in this case the presence of glyphosate in the medium significantly reduced the yield of soluble protein, resulting in a lower yield of 5-F-Trp-SPSB2. Incorporation of fluorinated tryptophan can also be achieved in non-auxotrophic BL21 cells by supplying them (in the absence of tryptophan) with 5-fluoroindole, which is considerably cheaper that 5-fluorotryptophan [36]. Other methods for incorporation of fluorine labels into protein amino acids, either by expression or post-translationally, have been reviewed recently [37].

In the spectra of 5-F-Trp-SPSB2 in the absence of ligand in Figure 1 and Figure 2, there are shoulders at −44.6 and −51 ppm. Moreover, these shoulders shift and/or become better resolved upon binding of some ligands, as illustrated in Figure 1c,d and Figure 2b–f. This is in contrast to the small peak around −49.2 ppm, which arises from a small amount of denatured protein in the NMR sample (Figure 1 and Figure 2). We do not believe that these shoulders arise from impurities because their intensities are identical across different fractions from the final purification step, as illustrated in Figure S2. This is supported by the observations in Figure 1 and Figure 2 that they change upon ligand binding. Shoulders of slightly lower intensity were observed when labelling was carried out using 5-F-indole as the substrate rather than 5-F-Trp (our unpublished observations) but some variation in relative intensities was observed among spectra of different samples prepared with 5-F-Trp, so this comparison cannot be considered significant at this stage.

It is likely that these peaks arise from another conformer of SPSB2 in solution. ^1^H-NMR spectra of both unlabelled SPSB2 and 5-F-Trp-SPSB2 show some evidence of additional conformers affecting Trp residues, as indicated in Figure S3, although the relative intensities of the shoulders in the ^19^F spectra exceed those of the minor peaks observable by ^1^H-NMR. Notwithstanding this, it appears that both forms of SPSB2, major and minor, bind peptide with similar affinities as ITC results for EKDINNNVK binding to 5-F-Trp-SPSB2 show 1:1 stoichiometry, identical to that of unlabelled SPSB2 [31].

### 3.2. Ligand Binding to Apical Membrane Antigen 1 (AMA1)

AMA1 is a well-characterized antigen on the surface of the invasive forms of the apicomplexan parasites, including *Plasmodium*, the causative agents of malaria. AMA1 has been extensively studied as a malaria vaccine candidate [38], but recent insights into the structure and function of AMA1 have established the protein as a potential target for anti-malarial drugs [39]. AMA1 has an essential role in the invasion of host cells by these obligate intracellular parasites. Specifically, AMA1 forms a complex with another group of parasite proteins, the rhoptry neck (RON) proteins, which are inserted into the host cell plasma membrane by the parasite prior to invasion [40,41,42]. The AMA1-RON complex thereby links the invading parasite with its host, allowing invasion to proceed.

**Figure 3:**
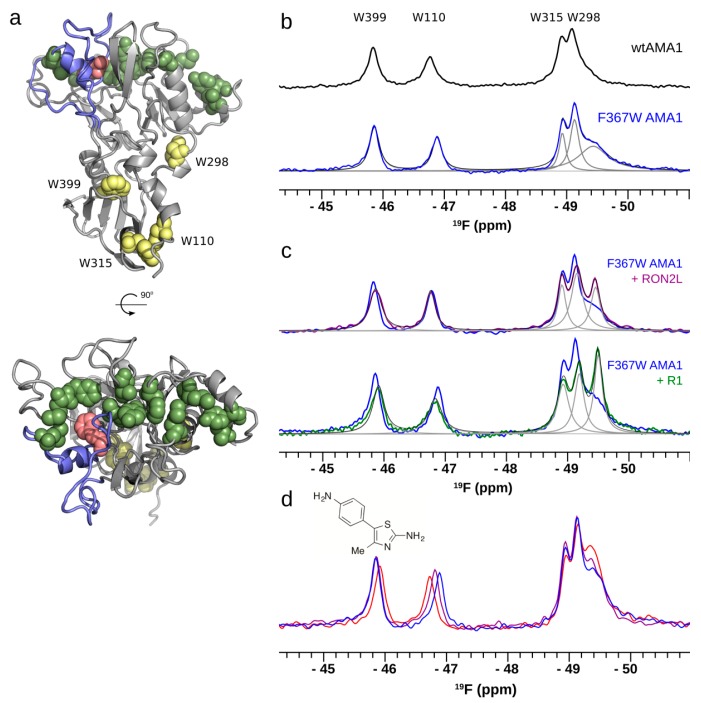
^19^F-NMR as a probe of ligand binding to AMA1. (**a**) The structure of AMA1 (domains I and II; PDB id 2Z8V) showing the RON2 binding site (green), the DII loop (blue), the site of the introduced 5-F-Trp 367 (red) and the four native Trp residues (yellow) (**b**) ^19^F-NMR spectrum of 5-F-Trp labelled F367W AMA1 (blue) contains signals from the four Trp residues in wt AMA1 (black; assigned by mutagenesis), plus an additional broad signal attributed to the introduced 5-F-Trp367. Deconvolution of the spectrum as the sum of five Lorentzian signals is shown (grey); (**c**) Addition of the peptide ligands RON2L (purple) or R1 (green) causes the 5-F-Trp367 resonance to become sharp; (**d**) A series of amino-thiophene fragments at 0 (blue), 1 (purple) and 3 mM (red) bind to AMA1 and cause concentration-dependent sharpening of the 5-F-Trp367 resonance. Samples contained ~100 µM 5-F-Trp F367W AMA1 in 20 mM sodium phosphate, pH 7.4, and spectra were recorded at 25 °C and a ^19^F frequency of 564 MHz without ^1^H decoupling [52].

Several monoclonal antibodies targeting AMA1 block the interaction with the RON complex and thereby inhibit parasite invasion [40,43,44]. Likewise, several peptides derived from phage-display or from AMA1’s binding partner in the RON complex, RON2, are inhibitory [45], establishing the AMA1-RON interaction as a potential therapeutic target [39,46]. X-ray crystallography has identified an extended hydrophobic cleft, flanked by flexible and polymorphic loops, which constitutes the RON2 binding site [47,48,49,50] (Figure 3a). The largest of these loops, the so-called DII loop, must be displaced in order to expose the full RON2 binding site, but this loop is not resolved in crystal structures of AMA1 in complex with RON2 or other ligands, leaving the details of this conformational change somewhat obscure.

To identify drug-like inhibitors of AMA1, we have recently screened our in-house fragment library using ligand-detected ^1^H-NMR and SPR [51]. From this screen we have identified and further developed a number of fragment series that bind the hydrophobic cleft, competing with the peptide ligand R1. We have used ^19^F-NMR to further define the binding site of these molecules, and to examine the conformational consequences of their interaction with AMA1. To do this, we introduced a ^19^F probe into the AMA1 DII loop by mutating Phe367 to Trp, and labelled the resulting construct with 5-F-Trp [52]. The ^19^F-NMR spectrum reveals five signals, corresponding to the four native Trp residues in AMA1, and the Trp introduced at residue 367 (Figure 3b). The linewidth of the signal from Trp367 is approximately 250 Hz, more than twice that of the native AMA1 Trp signals. This unusually broad linewidth indicates the presence of conformational exchange centered on the DII loop, consistent with our inability to complete the backbone assignments of this region of AMA1 [53] and with the fact that the DII loop is frequently not resolved in AMA1 crystal structures [49].

To assess the sensitivity of the 5-F-Trp367 signal to the conformational change that accompanies ligand binding, we tested the response of 5-F-Trp-AMA1[F367W] to binding of the peptide ligands RON2L (sequence DITQQAKDIGAGPVASCFTTRMSPPQQICLNSVVNTALS) and R1 (sequence VFAEFLPLFSKFGSRMHILK). A significant change in the ^19^F-NMR of 5-F-Trp-AMA1[F367W] was observed in the presence of both peptides, with the 5-F-Trp367 peak shifting upfield by 0.2 ppm and decreasing in linewidth to less than 90 Hz, becoming the sharpest peak in the spectrum (Figure 3c). The change in line-shape of the 5-F-Trp367 resonance may reflect a quenching of the exchange process that causes the 5-F-Trp367 resonance in the absence of peptide to be unusually broad, or a significant increase in the flexibility of W367 relative to the rest of AMA1. For several reasons, we favour the latter explanation: the chemical shift of the 5-F-Trp367 is identical in the presence of the two peptides, and is significantly sharper than the native Trp resonances of AMA1, consistent with the DII loop being displaced from the hydrophobic cleft and becoming highly flexible, as inferred from the crystallographic results.

We then tested the effect of members of our fragment series on the 5-F-Trp-AMA1[F367W] spectrum [52]. Of three series tested, one caused concentration-dependent sharpening of the 5-F-Trp 367 resonance, similar to that caused by the peptide ligands (Figure 3d). This result suggests that members of this series of elaborated fragments displace the DII loop in much the same way as the peptide ligands do, although it should be noted that the ligand shown in Figure 3d is a 2-aminothiazole, which we have shown in a separate study is a promiscuous binder that is not necessarily suitable as a starting point for an FBDD campaign [54]. In contrast, members of two other series cause no change to the 5-F-Trp-AMA1[F367W] spectrum, despite competing with R1 and therefore binding in the hydrophobic cleft. This suggests that these molecules bind at a distinct sub-site within the cleft, distal from the DII loop and accordingly do not displace the loop.

### 3.3. Competitive Binding

#### 3.3.1. Fluorinated Peptide Probe Targeting the iNOS Binding Site on SPSB2

**Figure 4:**
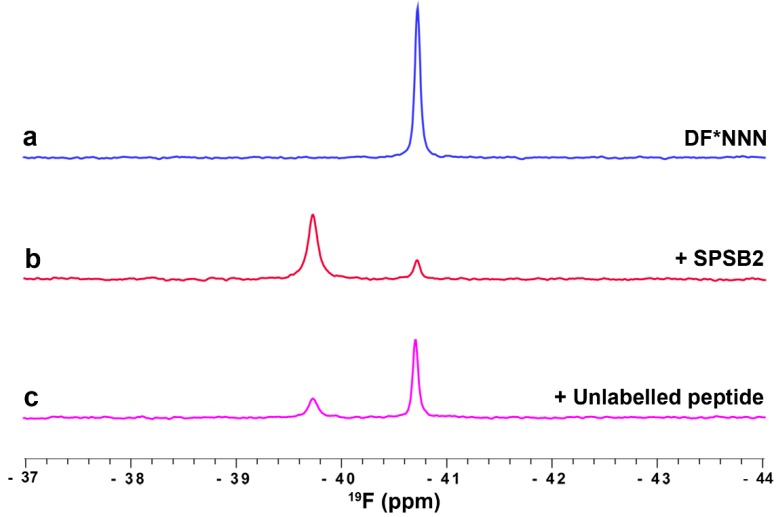
Fluorinated peptide ligand binding to SPSB2. ^19^F-NMR spectra of 100 µM fluorinated DF*NNN peptide in the absence (blue) and presence (red) of 100 µM SPSB2. Addition of 100 µM 13-residue iNOS peptide resulted in partial displacement of DF*NNN from the binding site (magenta). Spectra were recorded at 25 °C in 50 mM sodium phosphate, pH 7.4, 50 mM NaCl, at 564 MHz without ^1^H decoupling.

The binding of a fluorinated DINNN peptide to SPSB2 was explored by ^19^F NMR. The Ile residue of DINNN was chosen as a point of replacement with 4-fluoro-phenylalanine as mutants at this position have minimal effects on its binding to SPSB2 [26]. DF*NNN was shown to bind SPSB2 with a *K*_d_ of 3 ± 0.1 µM by SPR. Binding of the fluorinated peptide to SPSB2 was further explored by ^19^F-NMR (unpublished results; see Supplementary Information for experimental details). The ^19^F-NMR spectrum of 100 µM of DF*NNN gave a sharp peak at −40.7 ppm (Figure 4a). In the presence of 100 µM unlabelled SPSB2 (where the bound occupancy based on its *K*_d_ would be 98%) a new, broader peak appeared at −39.7 ppm (Figure 4b), corresponding to bound peptide. Subsequent addition of a longer, unlabelled peptide that binds strongly to SPSB2 (with a *K*_d_ of 4 nM) displaced most of the DF*NNN (Figure 4c).

It will be clear that experiments such as this provide a rapid means of identifying ligands that compete for the same binding site, with the DF*NNN acting in this example as the ‘spy’ molecule. Indeed, ligands such as DF*NNN, with a *K*_d_ for the target protein in the low µM range, represent a good choice as a displaceable ligand in the early stages of a fragment elaboration campaign, where ligands with µM to high nM *K*_d_ are being assessed. Although a quantitative titration was not undertaken in this example, it will be equally clear that the *K*_d_ of a non-fluorinated displacing ligand could be determined in this way provided the *K*_d_ of the fluorinated displaceable ligand is known. The equations relevant to this determination are described in the Supplementary Information [55]. Of course, *K*_d_ values can also be obtained directly for fluorine-containing ligands by plotting the chemical shift perturbation against ligand concentration [56].

#### 3.3.2. Profiling the AMA1 Binding Site Using Fluorinated R1 Peptide

**Figure 5:**
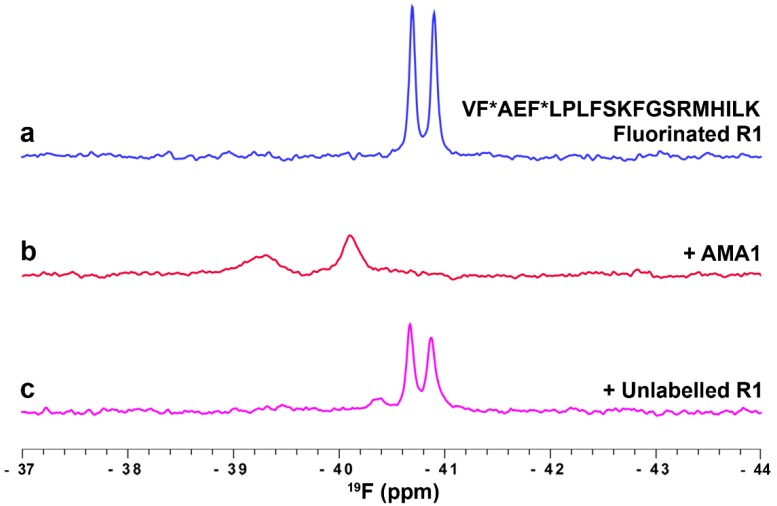
Monitoring ligand binding to AMA1 using a fluorinated R1 peptide probe. ^19^F-NMR spectrum of 10 µM 4-F-Phe R1 peptide in the absence (**a**) and presence (**b**) of 35 µM AMA1. 125 µM unlabelled R1 peptide was used to compete out the fluorinated R1 peptide from the hydrophobic cleft on AMA1 (**c**). Spectra were recorded at 30 °C in 20 mM sodium phosphate, pH 7.4, at 564 MHz without ^1^H decoupling.

R1, a 20-residue peptide identified from a phage-display library, binds to the hydrophobic cleft on AMA1 and blocks its interaction with RON2, as described above (Section 3.2). Molecules that block this interaction inhibit merozoite invasion of erythrocytes and are attractive leads in the development of novel anti-malarials. We have applied ^19^F-NMR spectroscopy to detect ligands binding specifically to the hydrophobic cleft of AMA1 (unpublished results; see Supplementary Materials for experimental details). A synthetic R1 peptide with Phe2 and Phe9 residues substituted with 4-fluoro-phenylalanine was used as the fluorinated reporter ligand in our studies. The ^19^F-NMR spectrum of fluorinated R1 displayed two sharp peaks at −40.7 and −40.9 ppm corresponding to the two fluorinated residues, Phe2 and Phe9 (Figure 5a). Significant changes in chemical shifts and line widths were observed in the ^19^F-NMR spectrum of R1 in the presence of AMA1, reflecting the high affinity interaction of R1 and AMA1 (Figure 5b). Having established the sensitivity of ^19^F-NMR in detecting ligand binding to the hydrophobic cleft of AMA1, we further explored the utility of fluorinated R1 probe in a ^19^F-NMR competition assay. A 12-fold molar excess of unlabelled R1 peptide was used in this assay to compete out the bound fluorinated R1 peptide resulting in the reappearance of sharp peaks at −40.7 and −40.9 ppm corresponding to free fluorinated R1 (Figure 5c). This assay is particularly useful in detecting ligands that target the hydrophobic cleft of AMA1 and thus provides an efficient way of evaluating inhibitors of the AMA1-RON2 interaction.

Although the examples above (Figure 4 and Figure 5) illustrate the application of ^19^F-NMR to characterising peptide-protein interactions rather than the interactions of elaborated fragments with proteins, both of the proteins exemplified, SPSB2 and AMA1, have been the subject of FBDD campaigns and the peptides have been valuable in confirming the binding sites of fragments and elaborated fragments on the target proteins.

### 3.4. Ligand Binding Site Mapping

An attribute of ^19^F-NMR that has been highlighted in many of the studies referred to above is the sensitivity of the ^19^F chemical shift to its environment. This leads to the idea that the binding site of a fluorine-containing elaborated fragment on a target protein could be ‘mapped’ by creating a series of fluorine-containing ligands that would be expected to bind in the same way, and then monitoring the magnitude of the chemical shift change upon binding. Systematic fluorine substitution around an aromatic ring, for example, could be undertaken to establish which positions are most perturbed by protein binding [18,21].

Figure 5 also exemplifies this in the case of a fluorinated peptide. In the free R1 peptide the two 4-F-Phe resonances have slightly different chemical shifts, although both are close to the random coil shift for 4-F-Phe (Figure S4). However, upon binding to AMA1, the chemical shift difference between the two resonances increases and the further downfield resonance is broader. We infer that the further downfield and broader peak in Figure 5b arises from Phe5 and the less shifted and sharper peak from Phe2 on the basis of mutational data on R1 [57,58] and the crystal structure of the R1-AMA1 complex [59], in which Phe5 makes more significant contact with AMA1 than does Phe2. Indeed, Phe2 can be omitted in truncated analogues of R1 without significant loss of binding affinity [57,58], whereas Phe5 is essential. Intriguingly, the resonance of Phe2 is still significantly shifted and broadened even though it makes little contribution to binding, providing a further example of the prevalence of transient or ‘fuzzy’ interactions in peptide-protein interactions [60] and of the sensitivity of ^19^F-NMR in mapping such interactions.

## 4. Conclusions

In this article we have reviewed various applications of ^19^F-NMR to the characterization of ligand-protein interactions, highlighting in particular its utility at all stages of the fragment-based drug discovery pipeline. We have further exemplified some of the attributes of ^19^F-NMR with unpublished examples of our own studies on ligand-protein interactions relevant to FBDD targets. The simplicity and low cost of ^19^F labelling of both ligands and target proteins, the high sensitivity of ^19^F for NMR detection, and the exquisite sensitivity of ^19^F chemical shifts and linewidths to ligand binding all make it a valuable approach in FBDD, and we anticipate that it will continue to contribute to the progression of fragments to drugs.

## Supplementary Materials

Supplementary materials can be accessed at: http://www.mdpi.com/1420-3049/21/7/860/s1.
