# Supplementary material for: Applications of 19F-NMR in Fragment-Based Drug Discovery
Source: Molecules. 2016 Jul 16;21(7):860. doi: 10.3390/molecules21070860 (PMC6273323; doi:10.3390/molecules21070860)
Supplement: Supplementary file 1 [file molecules-21-00860-s001.pdf]

Supplementary Materials: Applications of ^{19}F-NMR in Fragment-Based Drug Discovery

Raymond S. Norton, Eleanor W. W. Leung, Indu R. Chandrashekaran and Christopher A. MacRaild

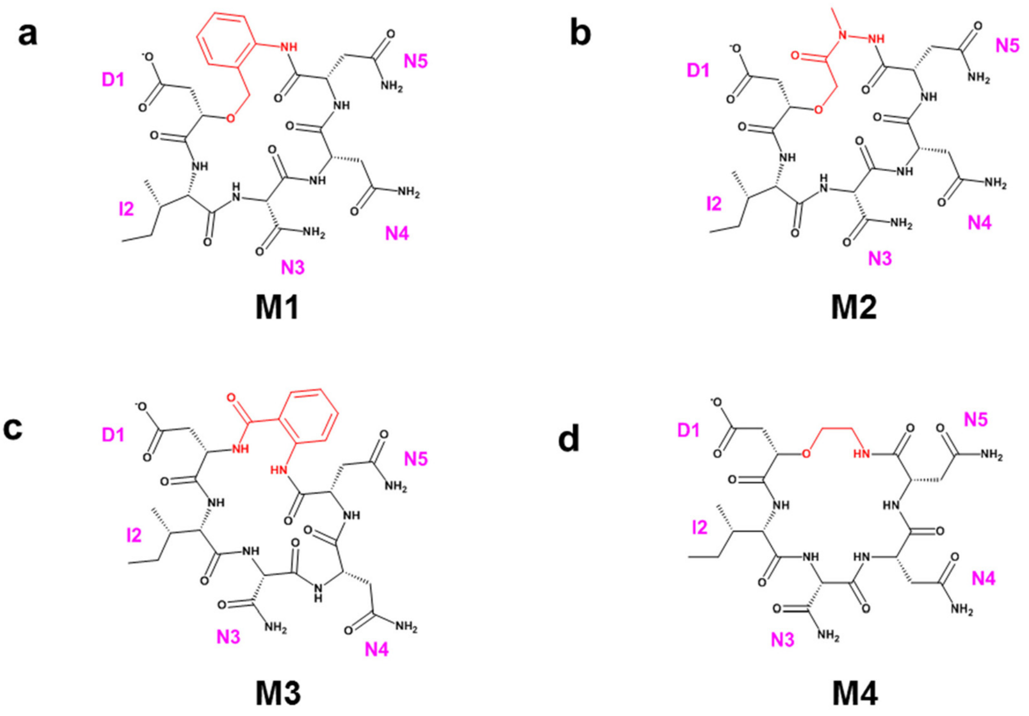

Figure S1. Chemical structures of four peptidomimetics (**a**) **M1**; (**b**) **M2**, (**c**) **M3** and (**d**) **M4**, with respective linker residues shown in red bridging the *N*- and *C*- termini of the DINNN peptide. Linker residues for **M1**, **M2**, **M3** and **M4** are benzyl, hydrazine, antranilamide and ethyl, respectively.

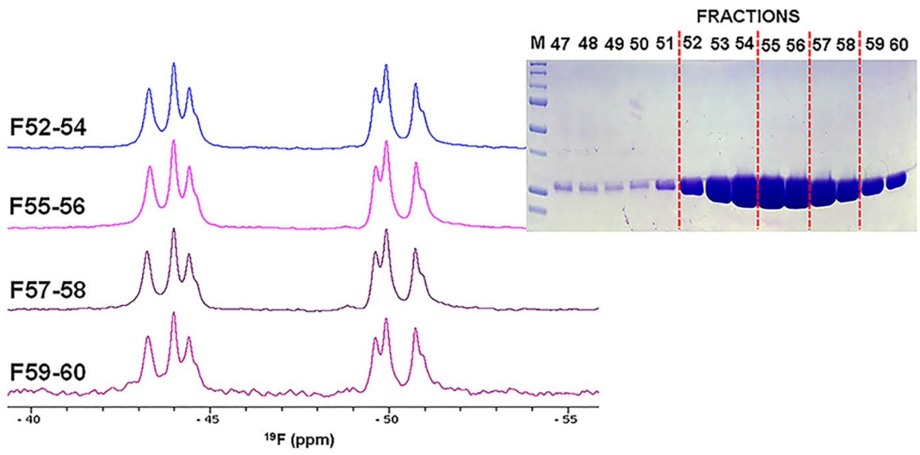

Figure S2. ^{19}F-NMR spectra of 5-F-Trp-SPSB2 samples prepared from different fractions of the size exclusion purification step. The intensities of the shoulders at −44.6 and −51 ppm are identical within experimental error across all fractions, indicating that these shoulders do not arise from impurities in the sample. ^{19}F-NMR spectra were recorded at 30 °C in 50 mM sodium phosphate, pH 7.4, 50 mM NaCl, 2 mM DTT, 2 mM EDTA, 0.02% sodium azide, at 564 MHz without ^{1}H decoupling on a Bruker Avance 600 spectrometer equipped with a cryoprobe tuned to ^{19}F.

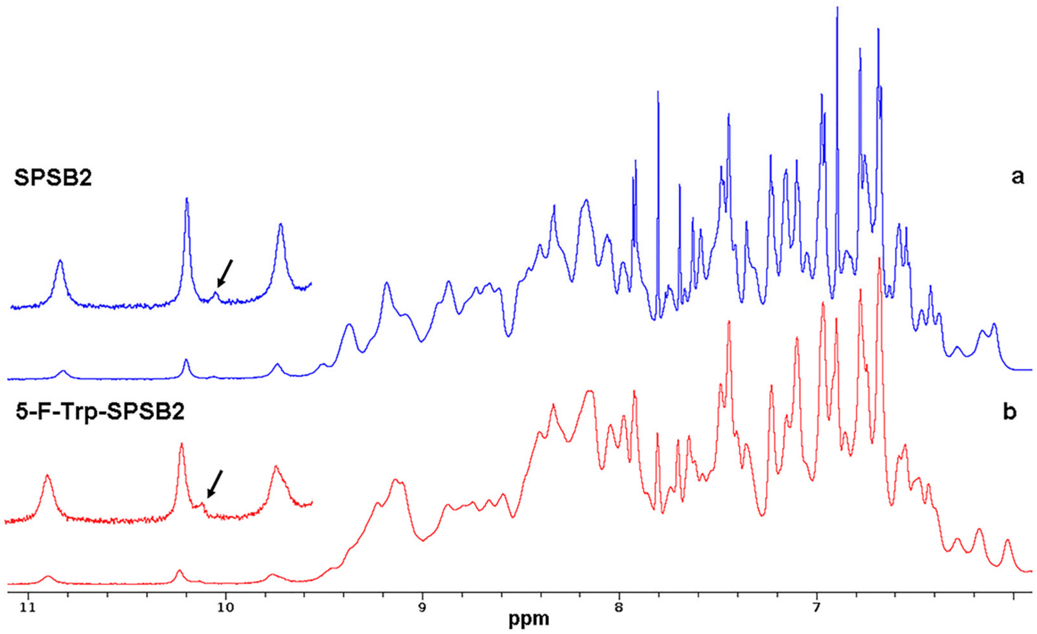

Figure S3. Aromatic and NH regions of the ^{1}H-NMR spectra of (**a**) SPSB2 and (**b**) 5-F-Trp-SPSB2 samples. Insets show vertical expansions of the indole NH peaks from Trp residues. The small peak at 10.06 ppm in the spectrum of SPSB2 (**a**) indicates that this Trp already experiences more than one environment in the unlabelled protein. In the spectrum of 5-F-Trp-SPSB2 (**b**) this minor peak is similar to or slightly larger than in the unlabelled protein. Spectra were recorded at 25 °C with 100 μM SPSB2 or 5-F-Trp-SPSB2 in 50 mM sodium phosphate, pH 7.4, 50 mM NaCl, 2 mM EDTA, 2 mM DTT and 0.02% sodium azide.

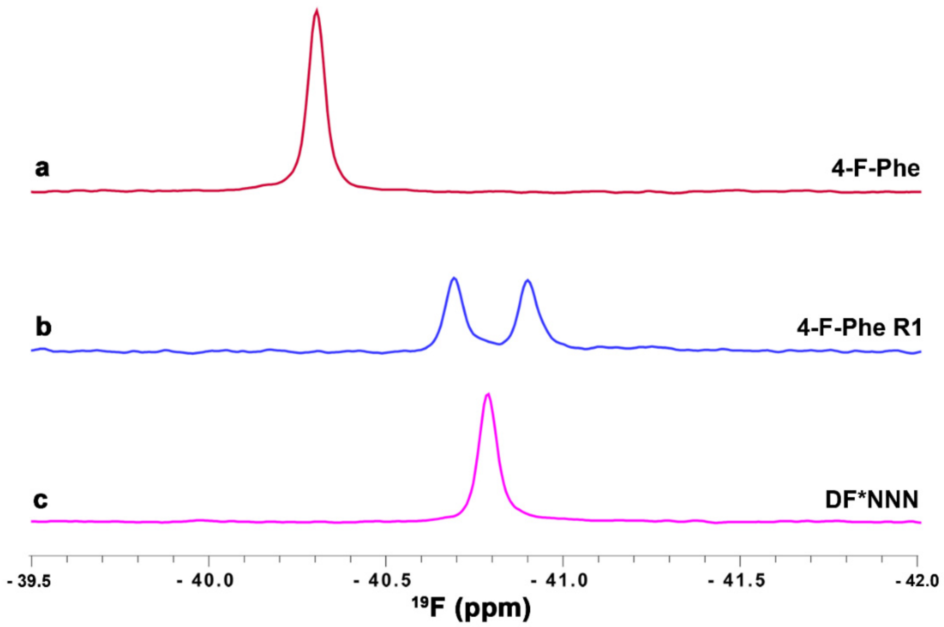

Figure S4. Comparison of ^{19}F-NMR spectra of 4-F-Phe and fluorinated peptides. (**a**) ^{19}F-NMR spectrum of 100 μM of the free amino acid 4-F-Phe (red); (**b**) 10 μM 4-F-Phe R1 peptide (blue) and (**c**) 50 μM 4-F-Phe DFNNN peptide (magenta). Spectra were recorded at 30 °C in 20 mM sodium phosphate, pH 7.4, at 564 MHz without ^{1}H decoupling.

1. NMR Spectroscopy

All NMR experiments were acquired on a Bruker Avance spectrometer (Billerica, MA, USA) operating at a ^{1}H frequency of 600 MHz using a TCI triple-resonance cryoprobe. One-dimensional ^{19}F- NMR spectra were acquired at 564 MHz with the ^{1}H channel tuned for ^{19}F. Spectra were recorded without ^{1}H decoupling, which is often the case for ^{19}F spectra recorded on conventional probes as opposed to those specifically designed for ^{19}F detection. A synthetic R1 peptide with Phe2 and Phe9 residues substituted with 4-fluoro-phenylalanine was sourced commercially (Purar Chemicals, Doncaster East, VIC, Australia). Samples contained 10 µM fluorinated R1 peptide in 20 mM sodium phosphate, pH 7.4, with 0.005% TFA as an internal reference for ^{19}F chemical shift. Lyophilised AMA1 was added to the sample to obtain a peptide: protein ratio of 1: 3.5. 125 µM of unlabelled R1 peptide was added to the mixture to displace the bound fluorinated R1 peptide. Spectra were recorded at 30 °C with a spectral width of 75.5 ppm and 1K scans with a relaxation delay of 1.5 s. Prior to Fourier transformation, the FIDs were processed with an exponential function using 20 Hz line broadening.

Fluorinated DFNNN peptide with Phe2 substituted with 4-fluoro-phenylalaine was sourced commercially (Purar Chemicals). ^{19}F-NMR spectra of 100 µM fluorinated DF*NNN peptide in 50 mM sodium phosphate, pH 7.4, 50 mM NaCl with 0.005% TFA as an internal reference for ^{19}F chemical shift. 100 µM SPSB2 was added to the sample to obtain a 1:1 peptide:protein ratio. For the competition experiment, 100 µM unlabeled 13-residue iNOS peptide was added to the mixture to displace the bound fluorinated DFNNN peptide. Spectra were recorded at 25 °C with a spectral width of 100 ppm and 1 K scans with a relaxation delay of 1.5 s. Spectra were processed with an exponential function using 20 Hz line broadening.

2. Ligand Competition Experiments

The ligand competition experiments are performed in the presence of a low-affinity fluorinated 'spy' molecule (with a K_D in the low µM range) and a non-fluorinated competing molecule (see reference 55 in the main text). The protein bound fraction (pb) of the 'spy' molecule can be derived using the equation:

$$p\text{b} = \frac{[E_T] + [L_T] + K_\text{D} - \sqrt{[E_T] + [L_T] + K_\text{D} - 4[E_T][L_T]}}{2[L_T]} \tag{1}$$

where [E_T] and [L_T] are the total protein and ligand concentrations, respectively, and K_D is the dissociation constant of the 'spy' molecule derived from ITC, SPR or NMR titration measurements.

The % displacement of the 'spy' molecule in the presence of a competing molecule can be calculated using the equation:

$$\%\ \text{Displacement} = 100 \times \left\{1 - \frac{(p\text{b})+}{(p\text{b})-}\right\} \tag{2}$$

where (pb)+ and (pb)− are the bound fraction of 'spy' molecule in the presence and absence of the competing molecule.

The apparent K_D (K_D^{app}) of the 'spy' molecule in the presence of the competing molecule is calculated using:

$$K_D^{app} = \frac{[E_T][L_T] - [E_T][EL] + [EL]^2 - [L_T][EL]}{[EL]} \tag{3}$$

where [E_T] and [L_T] are the total protein and ligand concentrations, respectively, and [EL] is the concentration of the protein-ligand complex in the presence of the competitor, which can be derived using $(p\text{b})+ = \frac{[EL]}{[L_T]}$.

The dissociation constant (K_I) of the competing molecule can be derived using the equation:

$$K_I = \frac{[I]K_D}{K_D^{app} - K_D} \quad (4)$$

where [I] is the concentration of the competing molecule, K_D is the true dissociation constant of the 'spy' molecule and K_D^{app} is the apparent dissociation constant of the 'spy' molecule in the presence of the competing molecule.
